# Supplementary material for: BE3 is the major branching enzyme isoform required for amylopectin synthesis in Chlamydomonas reinhardtii
Source: Front Plant Sci. 2023 May 31;14:1201386. doi: 10.3389/fpls.2023.1201386 (PMC10264815; doi:10.3389/fpls.2023.1201386)
Supplement: Supplementary file 1 [file DataSheet_1.pdf]

Name	Primer Sequences	Amplicon size and sequence targeted	Annealing Temp. Additives
**GENOTYPING**			
BE1-1F	5'-AGGTTGCCTGCATACCTCAC-3'	1398bp *BE1* locus	58°C 5% DMSO
BE1-1R	5'-TTACACGAGCAACAGGCAAC-3'		
BE1-2F	5'-GAATATGGCGGGAAAAGGAT-3'	1341bp *BE1* locus	55°C
BE1-2R	5'-GGTGCTTGACTGCTCGTGTA-3'		
BE2-1F	5'-CACAATGCCGTCCTTATCCT-3'	1335bp *BE2* locus	58°C 5% DMSO
BE2-1R	5'-AGTAGACACGGGGAGTGTGG-3'		
BE2-2F	5'-CCTCCCACACACACAAAGTG-3'	1139bp *BE2* locus	60°C 5% DMSO
BE2-2R	5'-CCACACTCCCCGTGTCTACT-3'		
BE3-1F	5'-TGCACGAGGGTAAGGGTAAG-3'	1153bp *BE3* locus	58°C 5% DMSO
BE3-1R	5'-AGAACGGTGGGCATAGACAC-3'		
BE3-3F	5'-ATGCAGCTTGGCAAGTTTCACC-3'	1770bp *BE3* locus	58°C 5% DMSO
BE3-3R	5'-CCCTAAAAGCTTCCCAACCATG-3'		
**RT-PCR**			
BE1RTF	5'-GCGACTACAACGAGTATCTG-3'	319bp *BE1* cDNA	60°C
BE1RTR	5'-CAGCCGGAACGCAATGGTCTG-3'		
BE2RTF	5'-GACTGCCATCACACACACGC-3'	590bp *BE2* cDNA	60°C
BE2RTR	5'-CGCTGCTCAGAACAATCTTG-3'		
BE3RTF	5'-CCACCTGGACAAGGCGTTCGGCTAC-3'	372bp *BE3* cDNA	60°C
BE3RTR	5'-GAGAAGTAGGGGCCCAGGTCC-3'		
PhoBRTF	5′-GCATGTTCCGCCAGACCA-3'	736bp *PHOB* cDNA	60°C
PhoBRTR	5′-TGCAGGAAGCGCCAGTTGA-3'		
**PRODUCTION OF RECOMBINANT PROTEINS**			
GATBE1F	5'-CACCGCGTCAACTGCCGCGCCACTG-3'	2263 bp complete *BE1* cDNA sequence without transit peptide	68°C G/C buffer
GATBE1R	5'-CTACACCACTTCGTCGCCGCTG-3'		
GATBE2F	5'-CACCGCTGCGAGTGCATACGCTGGGAG-3'	2488 bp complete *BE2* cDNA sequence without TP	70°C
GATBE2R	5'-CTACCTTGCAAAGTAGGGCCCCAG-3'		
GATBE3F	5'-CACCGCTGCTGCTCCGGGAGAGAAGC-3'	2524 bp complete *BE3* cDNA sequence without TP	68°C
GATBE3R	5'-TTAGCGGGAGAAGTAGGGGCCCAG-3'		

Supplemental Table 1: List of the primers used in this study. The amplification was performed following the manufacturer recommendations, PCR additives and annealing temperatures used are indicated in the right column. The sequences in red were introduced to allow the directional cloning of the Chlamydomonas cDNAs in the pENT-D-Topo vector creating a gateway entry vector for subcloning in pET300.
