# Supplementary material for: BE3 is the major branching enzyme isoform required for amylopectin synthesis in Chlamydomonas reinhardtii
Source: Front Plant Sci. 2023 May 31;14:1201386. doi: 10.3389/fpls.2023.1201386 (PMC10264815; doi:10.3389/fpls.2023.1201386)
Supplement: Supplementary file 2 [file DataSheet_2.pdf]

	CrBE1	CrBE2	CrBE3	OsBEI	OsBEIIa
CrBE2	54 / 67				
CrBE3	53 / 67	71 / 80			
OsBEI	56 / 71	53 / 67	53 / 68		
OsBEIIa	54 / 69	62 / 75	55 / 69	58 / 73	
OsBEIIb	54 / 69	63 / 76	60 / 73	58 / 72	77 / 83

Supplementary Figure S1. Percent identities and similarities for *Chlamydomonas* and rice BE isoforms.

Sequence identities (top left) and similarities (bottom right) for each *Chlamydomonas* and rice BE isoforms are displayed in percentages and were deduced from the alignment of the corresponding protein sequences.

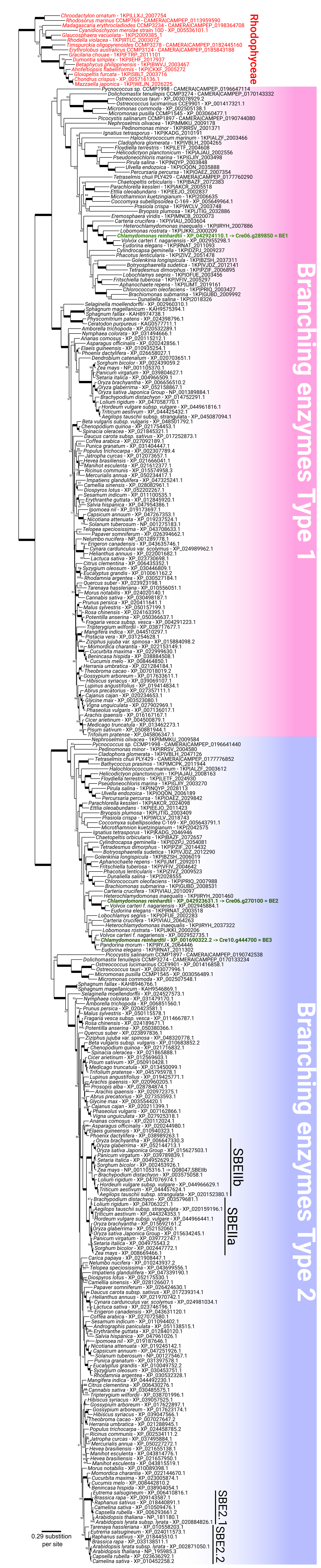

Supplementary Figure S2. Maximum likelihood consensus phylogenetic tree of starch branching enzymes in algae and plants.

The tree was reconstructed with IQtree using the model LG+C20+Γ and 1000 ultrafast bootstrap replicates as described in Material and Methods in the manuscript main text. Ultrafast-bootstrap branch support values are indicated, those having 100% bootstrap support are represented as thick lines. The rhodophyceae clade was used as an outgroup. A molecular distance scale is provided on the bottom left. Protein references are GenBank Accession numbers, except for: CAMERA which are data IDs provided by the MMETSP project (Keeling et al., 2014), and 1KP which are data IDs provided by the 1000 plant genomes project (Matasci et al., 2014). Chlamydomonas BE proteins are colored in green.

Keeling, P.J. et al. (2014). The Marine Microbial Eukaryote Transcriptome Sequencing Project (MMETSP): Illuminating the Functional Diversity of Eukaryotic Life in the Oceans through Transcriptome Sequencing. PLoS Biol. 12: e1001889.

Matasci, N. et al. (2014). Data access for the 1,000 Plants (1KP) project. Gigascience 3: 17.

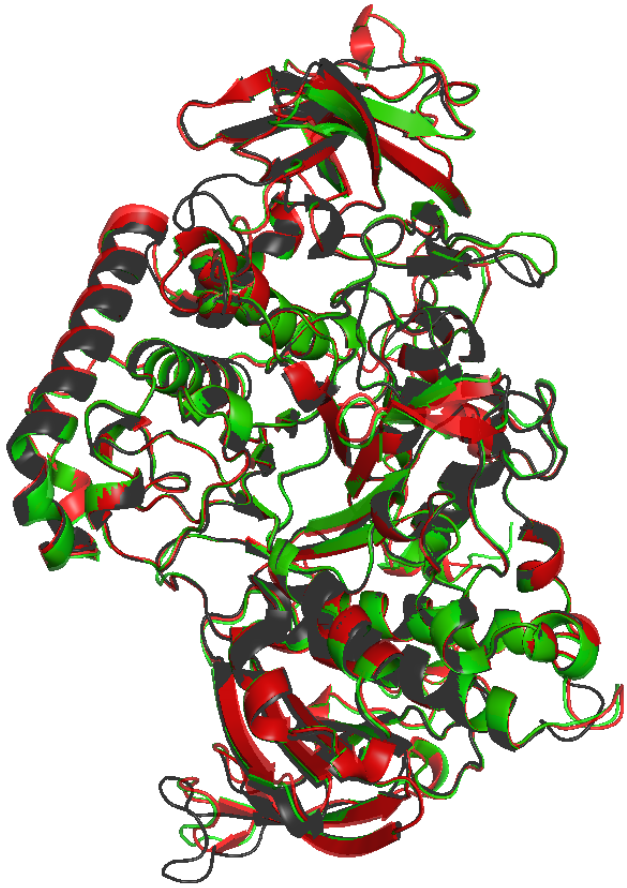

Supplementary Figure S3 : Overlay of Alphafold structures of Branching Enzymes from *Chlamydomonas reinhardtii*.

CrBE1 (black), CrBE2 (red) and CrBE3 (green) are represented as cartoons. The nature of the three domains, characteristics of the GH13 family is defined on the right panel.

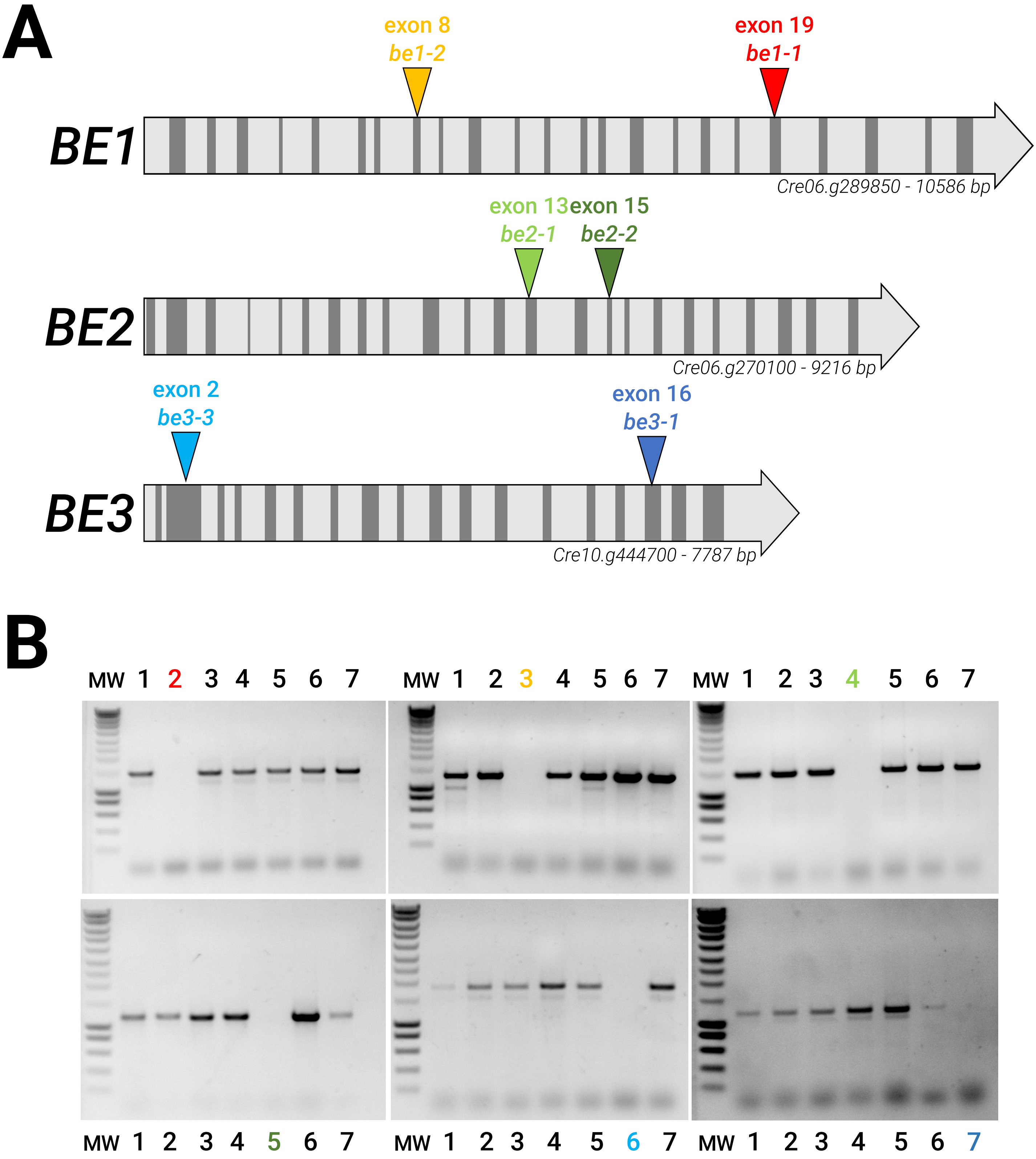

Supplementary Figure S4: Characterization of the genomic deletion in the *be* mutant strains.
(A) Schematic representation of the BE loci and position of the insertional mutation in each mutant allele are indicated. (B) Amplification of the genomic region covering the putative insertion of the paromomycin K7 in the algal genome for each mutant allele was performed using specific primers listed in supplemental table 1. MW: Eurogentec Smartladder. The genomic DNA used for amplification were extracted from the wild-type CC5325 (1), the *be1-1* (2), *be1-2* (3), *be2-1* (4), *be2-2* (5), *be3-1* (6), and *be3-3* (7) strains.

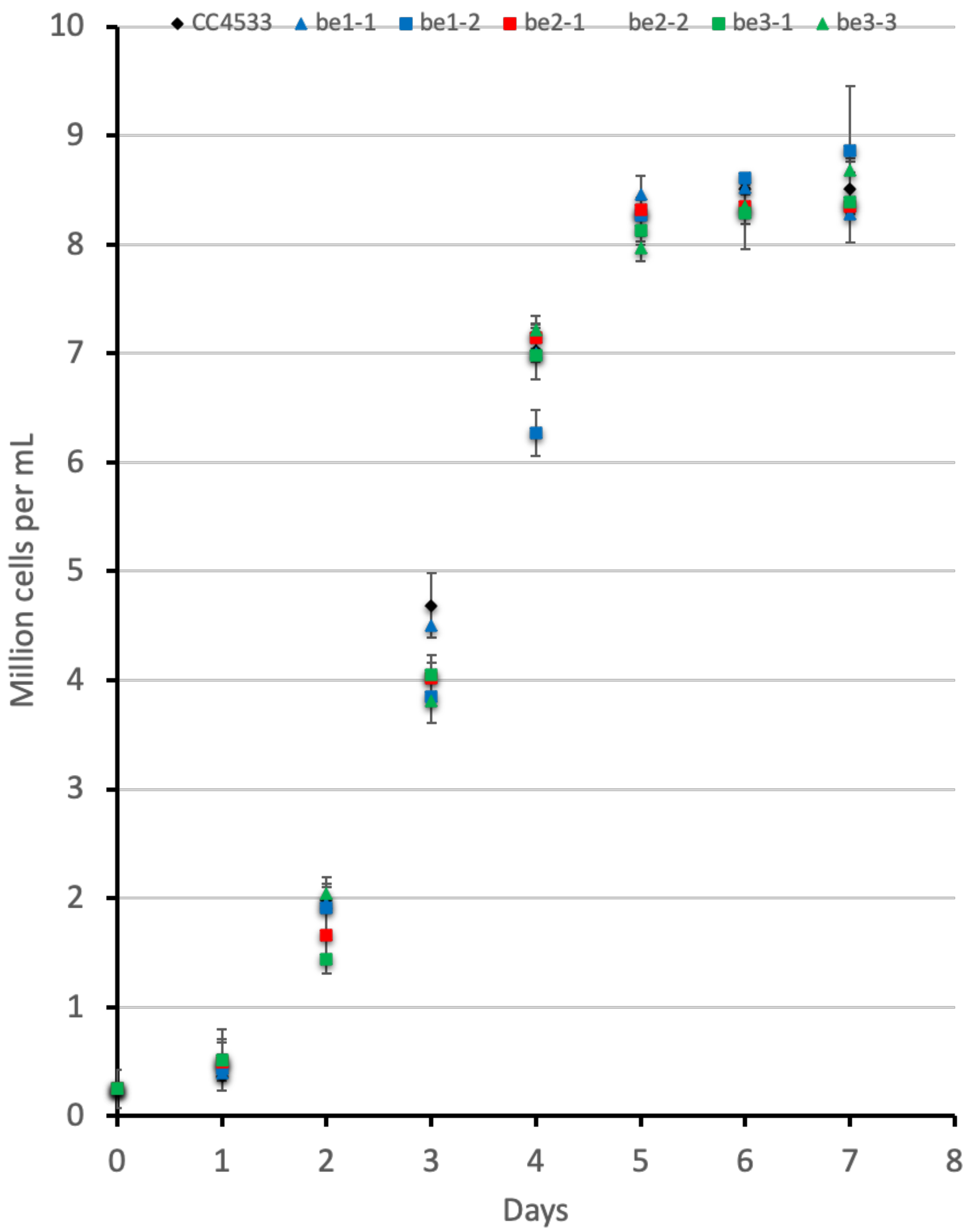

Supplementary figure S5: Growth curves of *Chlamydomonas* wild type and *be* mutant strains. *Chlamydomonas* strains were cultivated in one liter of TAP medium under continuous light. The cultures were inoculated at a density of 2.5 10^5 cells per ml and cell numbers were monitored with a Beckman Multisizer 4. The results are displayed as means ± SD of three independent cultures.

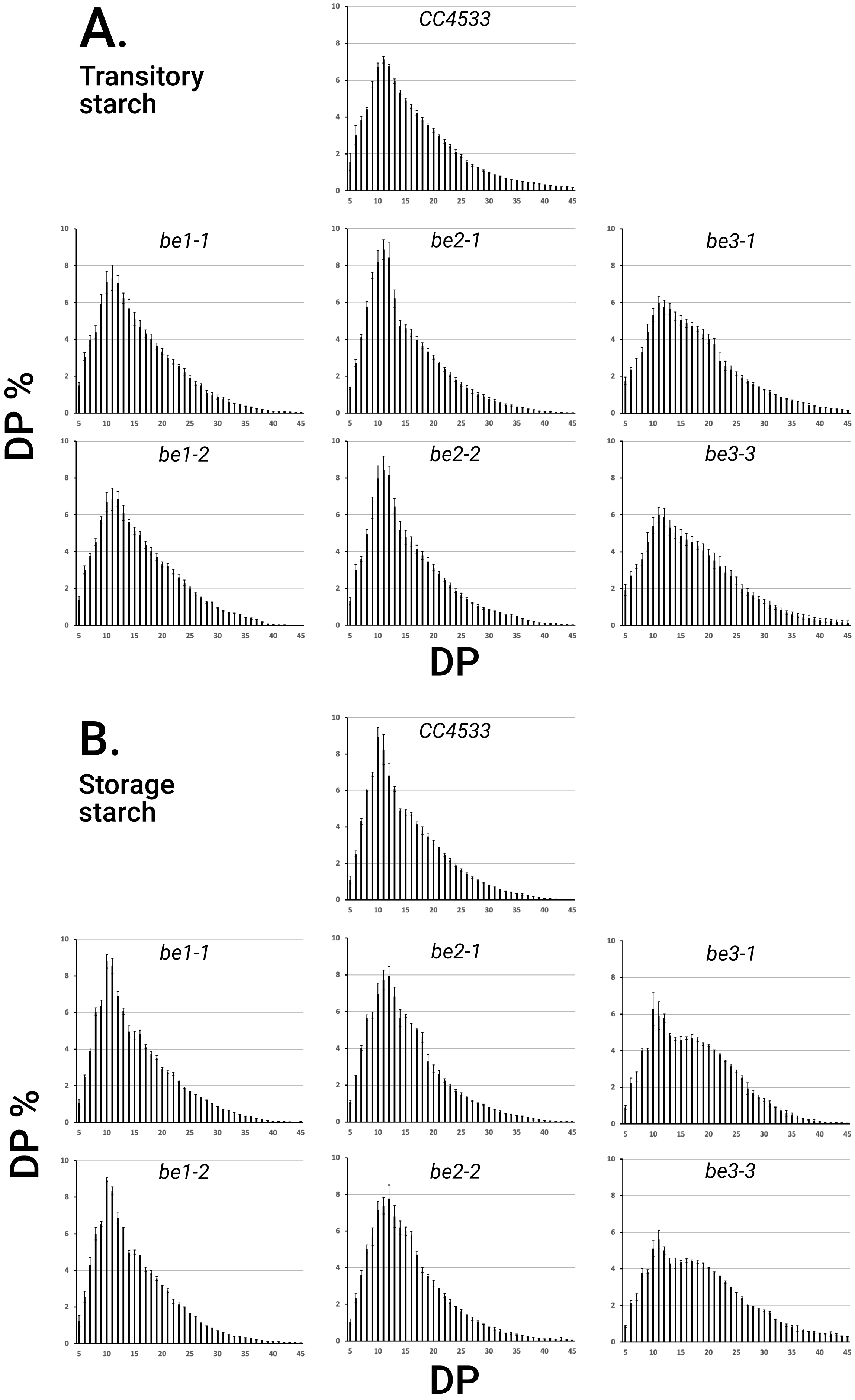

Supplementary figure S6: Chain length distribution of wild-type and mutant amylopectins.
Distribution of chain lengths of wild-type and mutant amylopectin after isoamylase-mediated debranching was confirmed by capillary electrophoresis of APTS-labeled glucans. Amylopectins were purified from transitory (a) or storage (b) starch through gel permeation chromatography. The results are displayed as the means ± SD of three independent biological samples. DP: degree of polymerisation, DP%: molar percentage of each chain.
